# Chitosan Dermal Substitute and Chitosan Skin Substitute Contribute to Accelerated Full-Thickness Wound Healing in Irradiated Rats

**DOI:** 10.1155/2013/795458

**Published:** 2013-11-13

**Authors:** Abu Bakar Mohd Hilmi, Ahmad Sukari Halim, Hasnan Jaafar, Abu Bakar Asiah, Asma Hassan

**Affiliations:** ^1^Reconstructive Sciences Unit, School of Medical Sciences, Universiti Sains Malaysia, 16150 Kelantan, Malaysia; ^2^Craniofacial Laboratory, School of Dental Sciences, Universiti Sains Malaysia, 16150 Kelantan, Malaysia

## Abstract

Wounds with full-thickness skin loss are commonly managed by skin grafting. In the absence of a graft, reepithelialization is imperfect and leads to increased scar formation. Biomaterials can alter wound healing so that it produces more regenerative tissue and fewer scars. This current study use the new chitosan based biomaterial in full-thickness wound with impaired healing on rat model. Wounds were evaluated after being treated with a chitosan dermal substitute, a chitosan skin substitute, or duoderm CGF. Wounds treated with the chitosan skin substitute showed the most re-epithelialization (33.2 ± 2.8%), longest epithelial tongue (1.62 ± 0.13 mm), and shortest migratory tongue distance (7.11 ± 0.25 mm). The scar size of wounds treated with the chitosan dermal substitute (0.13 ± 0.02 cm) and chitosan skin substitute (0.16 ± 0.05 cm) were significantly decreased (*P* < 0.05) compared with duoderm (0.45 ± 0.11 cm). Human leukocyte antigen (HLA) expression on days 7, 14, and 21 revealed the presence of human hair follicle stem cells and fibroblasts that were incorporated into and surviving in the irradiated wound. We have proven that a chitosan dermal substitute and chitosan skin substitute are suitable for wound healing in full-thickness wounds that are impaired due to radiation.

## 1. Introduction

 Wound healing is a complex biological process that involves molecular and cellular responses. The function of wound healing is to rapidly and functionally produce skin layers and appendages that are free of scars and as physiologically fit as native skin. The reconstruction of skin after injury involves different types of wound healing depending on the wound classification. For example, skin with a superficial loss of the epidermis gradually heals over time without intervention. In a partial-thickness wound, the epidermis primarily heals by re-epithelialization, which is the resurfacing of a wound bed by neokeratinocytes. In a surgical setting, partial-thickness wounds heal by primary intention, also known as surgical wound healing. This type of healing employs *sutures, staples, glue,* or strips between both sides of the wound edge to close the wound bed. The major events in healing by primary intention are connective tissue deposition and re-epithelialization. There is no formation of granulation tissue or wound contraction [1].

Wounds with a full-thickness loss of the dermis and epidermis cannot be repaired by primary intention. In the case where a large amount of skin is removed or destroyed, a gap occurs in the wound or a nonviable wound margin is present. As a result, the wound edges cannot be approximated. The presence of a wound gap prevents re-epithelialization. In this case, wounds can only heal by grafting epidermis over the wounded area. In the absence of a graft, the wound will reepithelialize slowly and imperfectly via the ingrowths of cell from the wound edges, which leads to increased scar formation.

However, biomatrices may be an alternative to a graft. Biomatrices are made of biopolymers that are often resorbed or degraded in the body and are regularly used in therapeutic applications [2], especially in skin tissue engineering. With biomatrices, wound healing can be manipulated to produce more regenerative tissue than scar tissue [3]. Biomatrices or cell-biomatrix constructs create the optimal conditions for accelerated wound healing which result in less or no scar formation and easy handling for transplantation and replaces the use of skin grafts. In the current study, full-thickness wounds with impaired healing were treated with three biomaterials: a chitosan dermal substitute, a chitosan skin substitute, and duoderm CGF. Wounds were then histomorphometrically evaluated. We concluded that the chitosan dermal substitute and chitosan skin substitute contributed to the accelerated wound healing in irradiated rats. 

## 2. Materials and Methods 

This research was approved by the Animal Ethics Committee of Universiti Sains Malaysia. Approval code: USM/Animal Ethics Approval/2009/(44)(133). The animal procedures were conducted in accordance with the guidelines of the Animal Research and Service Centre, USM. 

### 2.1. Animal Radiation

Three-month-old male Sprague Dawley rats, weighing 300–350 g, were randomized into three groups based on postwound time frames of 7, 14 or 21 days (*n* = 5 each). The rats were housed in separate cages at 22°C–26°C and alternating 12-hour light and dark cycles in a controlled room. The rats were fed a standard laboratory diet and water *ad libitum.* Prior to radiation, the rats were anesthetized with an intramuscular injection of ketamine (100 mg/kg) and xylazine (20 mg/kg). The dorsal area was shaved and marked. Skin irradiations were carried out using the source-skin distance technique (SSD) with a 6 MV photon beam from a linear accelerator (Siemens Primus, Germany). A SSD at 100 cm with a gantry angle of 0°, a collimator 0°, and a radiation field size of 3 cm × 10 cm was performed on the dorsum of each rat. The dorsum of each rat was given a single dose of 10 Gy. A 1.5 cm tissue-equivalent bolus of material was placed over the dorsal area to bring the full radiation dose to the skin surface [4, 5].

### 2.2. Biomaterials

Three bio-templates for skin regeneration were used: a chitosan dermal substitute, a chitosan skin substitute, and duoderm CGF. The chitosan sponge was fabricated as previously described [6]. In this study, it is named as chitosan dermal substitute. It was produced from ultrapure medical-grade chitosan powders of prawn shell. Chitosan powders were irradiated with 10 kGy of gamma radiation to produce a molecular weight of 440,000 Daltons and dissolved in 0.5 M acetic acid which formed a solution. To obtain a sponge layer, the chitosan solution is poured into a 10 cm by 10 cm polytetrafluoroethylene (PTFE) mold, deep-frozen at −20°C for 24 hours and freeze drying for 20 hours. Duoderm CGF is commercially produced (Convatec, USA). It composed of gel and matrix layers. To fabricate the chitosan skin substitute, chitosan sponge matrices were seeded with human dermal fibroblasts at a density of 3 × 10^6^/cm^2^ for two weeks, followed by being co-cultured or one week with primary human hair follicle stem cells (HFSCs) at a density of 1 × 10^6^/cm^2^. The isolation of HFSCs was described previously [7]. Briefly, the dermis containing hair follicle was incubated in 0.1% (w/v) collagenase type I (Gibco, USA) before being cultured in CnT-07 medium (CellnTech, Switzerland). The coculture was performed using a combination of Dulbecco's Modified Eagle Medium/Ham's F12 (DMEM/F12) (Invitrogen, USA) and supplemented with 5% fetal bovine serum (FBS) (Invitrogen, USA) and CnT-07 medium at ratio of 1 : 2. After three weeks in culture, the chitosan skin substitute was harvested for wound healing experiments.

### 2.3. Wound Creation

Two months postradiation, three full-thicknesses wounds 1 cm by 1 cm in size were excised on the irradiated dorsum of each rat. Prior to incision, the rats were anesthetized with an intramuscular injection of ketamine (100 mg/kg) and xylazine (20 mg/kg). The fur on the dorsal skin was shaved. The shaved area was scrubbed and sterilized with 70% alcohol and then sprayed with povidone-iodine. Animals were placed on a heated surgery table and incisional wound was created using a sterile scalpel and blade (size 10). Each of the wounds was randomly covered with one of the three different types of biomaterials as mentioned above. 

### 2.4. Histomorphometrical Analysis

To determine the length of the epithelial tongue, the distance of the migratory tongue, and the scar size, a computer-based histological image analysis (*n* = 5) was performed using the Mirax Viewer (Zeiss, Germany). The scar size was measured between the gaps in granulation tissues as mentioned previously [8] with modification. The percentage of re-epithelialization was measured as the ratio of the neoepidermis and the wound area [9]. The percentage of the wound size was calculated as the ratio of the wound size at *day x* over the wound size at *day 0* [10]. 

### 2.5. Wound Assessment

The wound assessment was performed without removing the biomaterials or cleaning the debris from the wound bed during the healing process. Seven parameters were assessed on days 7, 14, and 21 using the following scoring system: infection (pus), 1 if absent or 2 if present; hematoma, 1 If absent or 2 if present; exudates, 1 if high, or 2 if intermediate, or 3 if low; odor, 1 if strong, or 2 if intermediate, or 3 if none; flexibility, 0 if not flexible or 3 if flexible; adherence of biomaterials, 0 if nonadherent or 3 if adhered strongly; and fluid accumulation on biomaterials, 0 if yes or 3 if no. Scoring was based on the wound edge because the wound bed was covered with permanent tissue-engineered biomaterials.

### 2.6. Dressing

Secondary wound dressings were performed using Hypafix (BSN Medical, Germany) and Tg fix (Lohmann & Rauscher, Germany). Prior to the dressing, the rats were anesthetized with inhaled isoflurane. To avoid attaching the biomaterials to the Hypafix which causes tears in the biomaterials after removing the Hypafix, gauze was used to cover the sticky side of a 1 cm by 1 cm piece of opsite flexi grid (Smith & Nephew, England) and this was applied over the biomaterial. Tg fix was used as a second layer of dressing before the wounds were covered with a bandage. The wound dressing was changed every three days.

### 2.7. Wound Evaluation

Rats in each group were sacrificed on postoperative days 7, 14, or 21 with an intramuscular injection of an over dose of ketamine (100 mg/kg) and xylazine (20 mg/kg). Images of the wounds were captured and measured before the wounds were excised. The excised area included the wound bed and intact skin and was then fixed with 10% formalin before histological analysis using H&E staining. The images of stained samples were captured, measured, and analyzed using a Mirax Desk Scanner (Zeiss, Germany). 

### 2.8. Immunofluorescence

Immunofluorescent staining was performed on paraffin-embedded sections. Sections were placed on hot plate for two hours, deparaffinized in xylene, and rehydrated using a graded series of 100, 95, 80, 70, and 50% ethanol followed by distilled water. Pretreatment was performed using a water bath containing a target retrieval solution (pH 9) (Dako, Denmark) at 98°C for 15 minutes. Evaluation of the human tissue engrafted into the wound bed was performed using mouse monoclonal antibodies against human HLA (1 : 100) (Abcam, UK) or rat K10 (1 : 500) (Abcam, UK) for evaluation of the proliferation of the neoepidermis. To block nonspecific antibody-antigen binding, sections were preincubated with 10% normal serum in tris-buffered saline (TBS) for 20 minutes. Incubation with a primary antibody was performed at 4°C overnight. Sections were incubated with a fluorescent goat polyclonal secondary antibody against mouse IgG at room temperature for 45 minutes. Nuclei were counter stained with DAPI. Slides were mounted with fluoromount-G (Southern Biotech, USA) and viewed under Axioplant2 fluorescent microscope (Zeiss, Germany). 

### 2.9. Statistics 

The data on physical observation are presented as the means ± SEM. The rest, an ANOVA was used for analyses. A significant difference was considered when *P* < 0.05. Bonferroni test was used to identify statistically significant differences between specific intergroup mean values.

## 3. Results 

**Figure 1:**
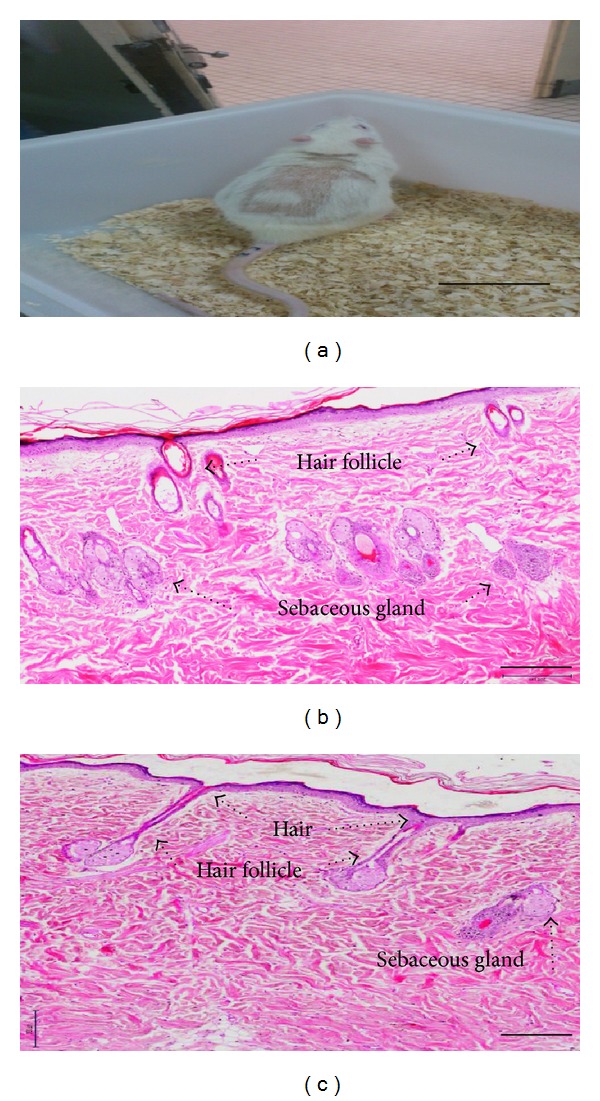
Radiation-induced temporary hair loss at dorsal area (a). Hematoxylin and Eosin (H&E) stained images of irradiated rat (b) and nonirradiated rat (c). Irradiated skin has no hair; anatomical and functional changes in hair follicles and sebaceous glands. Scale bar: (a) 3 cm, (b) and (c): 200 *µ*m.

**Figure 2:**
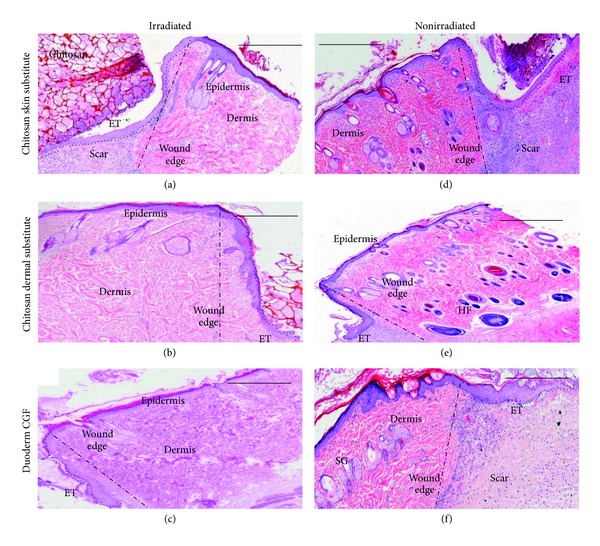
H&E stained images of irradiated and nonirradiated wounds on day 7. The square dotted line indicates the epithelial tongue (ET). The dash dotted line shows the area between wound edge and unwounded skin. Scale bar: 500 *µ*m. (SG: sebaceous gland, HF: hair follicle).

Postradiation symptoms in rats can be observed physically and histologically (Figure 1). During the proliferation stage at day 7, the migratory tongue distance (MT), the epithelial tongue (ET), and re-epithelialization were analyzed for both the irradiated and nonirradiated groups. The ET in irradiated wounds treated with a chitosan skin substitute was longer (1.62 ± 0.13 mm) than those in the irradiated wounds treated with a chitosan dermal substitute (1.22 ± 0.19 mm) or duoderm (1.14 ± 0.18 mm) (*P* = 0.20 and *P* = 1.0, resp.) In the nonirradiated wounds, the ET was longer after wounds were treated with a chitosan dermal substitute (1.23 ± 0.12 mm) compared to a chitosan skin substitute (1.19 ± 0.21 mm) or duoderm (1.11 ± 0.30 mm) (Figure 2) (*P* = 1.0).

**Figure 3:**
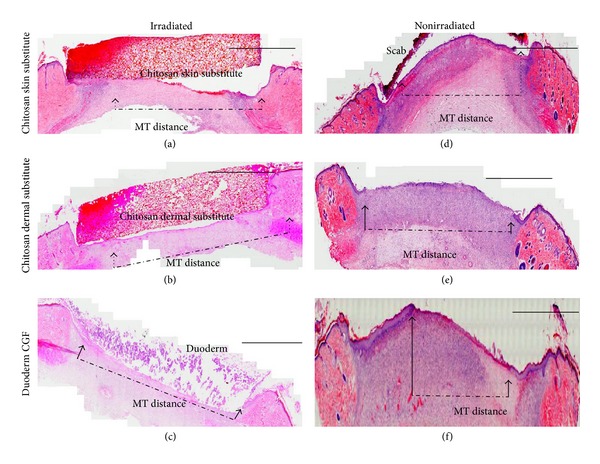
H&E stained images of irradiated and nonirradiated wounds on day 7. The arrow head indicates the epithelial tongue. The dash dotted lines show the outline of the migratory tongue (MT) distance. All biomaterials were adsorbed in wound bed in nonirradiated group ((d), (e), (f)). Scale bar: 200 *µ*m.

The MT distances in irradiated wounds treated with chitosan skin substitutes were shorter (7.11 ± 0.25 mm) than irradiated wounds treated with a chitosan dermal substitute (8.16 ± 0.26 mm) or duoderm (7.25 ± 0.47 mm) (*P* = 1.0 and *P* = 0.26, resp.). In nonirradiated wounds, the MT distances were shorter in wounds that were treated with duoderm (5.61 ± 0.71 mm) compared to the chitosan skin substitute (7.23 ± 0.47 mm) or the chitosan dermal substitute (5.90 ± 0.61 mm) (Figure 3) (*P* = 0.24 and *P* = 1.0, resp.). 

**Figure 4:**
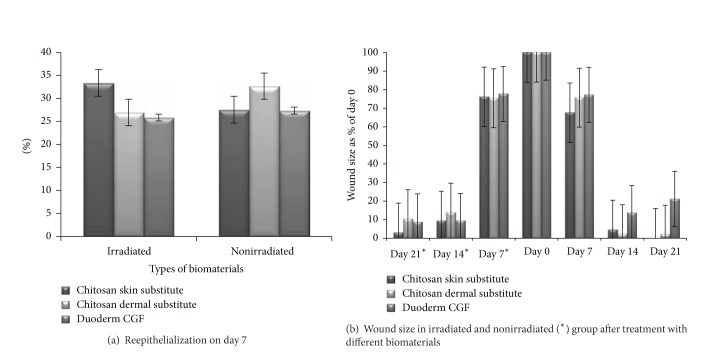
Reepithelialization of irradiated and nonirradiated wounds on day 7 (a) and full-thickness wound size evaluation (b).

There was greater re-epithelialization in the irradiated wounds treated with the chitosan skin substitute (33.2 ± 2.8%) than those treated with the chitosan dermal substitute (26.8 ± 4.8%) or duoderm (25.7 ± 3.7%) (*P* = 0.577 and *P* = 1.0, resp.). In nonirradiated wounds, the re-epithelialization was higher after wounds were treated with the chitosan dermal substitute (32.5 ± 3.1%) compared to the chitosan skin substitute (27.4 ± 4.1%) or duoderm (27.2 ± 6.3%) (*P* = 1.0) (Figure 4(a)). 

Between day 7 and day 21, nonirradiated wound sizes were decreased after coverage with skin substitutes. In irradiated wounds, the chitosan skin substitute was associated with a complete repair of the full-thickness wounds by day 21. Conversely, duoderm CGF was found to increase the wound size on day 21 compared to day 14. It was suggested that duoderm CGF cannot be used for long-term dressings for impaired healing (Figure 4(b)). Duoderm CGF can only be used for temporary primary dressing. 

**Figure 5:**
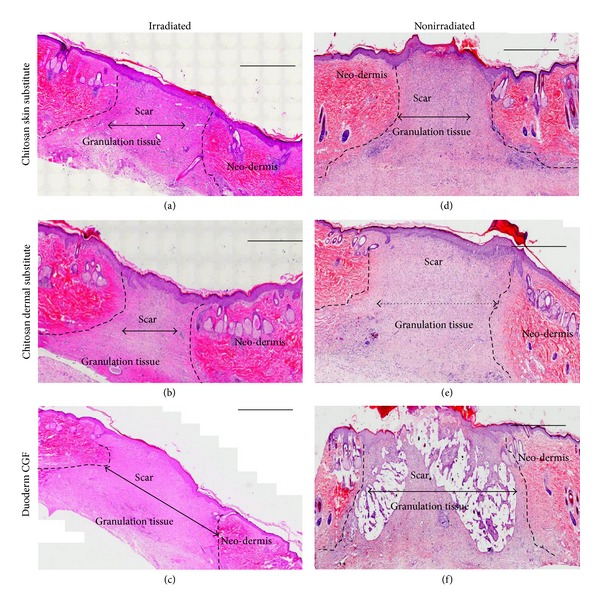
H&E stained images of irradiated and nonirradiated wounds on day 21. The double-headed arrows indicate the width of the scar. The dashed lines show the outline of the neodermis regeneration and granulation tissue. Scale bar: 1000 *µ*m.

During the remodeling stage, sampled on day 21, the chitosan dermal substitutes and chitosan skin substitutes significantly contributed to the acceleration of wound repair in impaired healing when compared to duoderm in terms of scar size (*P* = 0.039 and *P* = 0.023, resp.). The scar size was smaller in wounds treated with the chitosan dermal substitute (0.13 ± 0.02 cm) and the chitosan skin substitute (0.16 ± 0.05 cm). However, with duoderm, the scars were longer (0.45 ± 0.11 cm). In nonirradiated wounds, treatment with the chitosan skin substitute was associated with smaller scar sizes (0.18 ± 0.04 cm) than those in wounds treated with the chitosan dermal substitute (0.22 ± 0.04 cm) or duoderm (0.23 ± 0.08 cm) (Figure 5). However, the difference was not significant (*P* = 1.0). 

**Figure 6:**
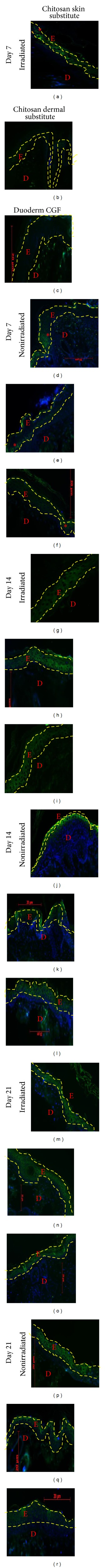
Expression of K10 along the migratory tongue. On day 7 postwound, there is no expressions in the irradiated rat ((b), (c)). However, the wound with chitosan skin substitute slightly expressed K10 (a with asterisk), and the nonirradiated rat showed similar results ((d), (e), (f)). K10 started to be slightly expressed in the irradiated rat on day 14 ((g), (h), (i)). K10 was highly expressed on day 14 in the nonirradiated rat ((j), (k), (l)) and on day 21 in the irradiated rat ((m), (n), (o)). The nonirradiated rats continuously expressed K10 on day 21 ((p), (q), (r)). Scale bar: 20 *µ*m. (E: epidermis, D: dermis).

**Figure 7:**
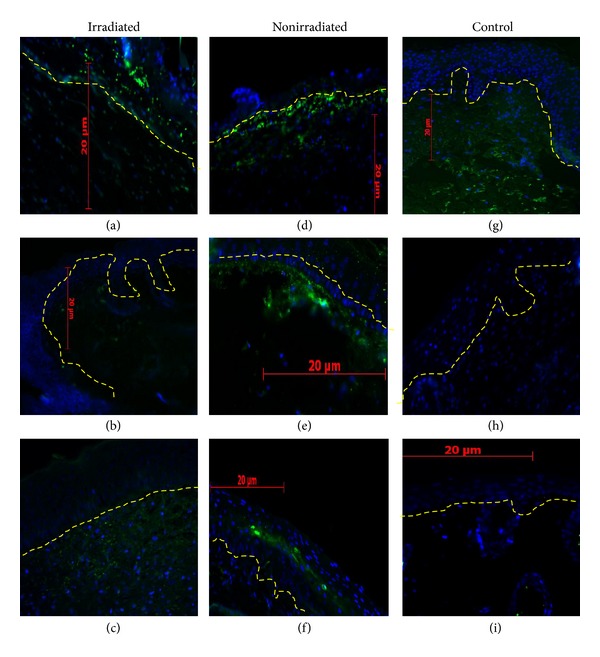
HLA (green fluorescence) was incorporated into wounds on days 7 ((a), (d)), 14 ((b), (e)), and 21 ((c), (f)). The positive control of human skin (g). The negative control of chitosan dermal substitute-treated wounds (h) and duoderm-treated wounds (i). The epidermis and dermis are demarcated via the dash dotted lines. Nuclei stained blue. Scale bar: 20 *µ*m.

K10 expression along the migratory tongue was assessed on days 7, 14, and 21 (Figure 6). The mode of expression was different between irradiated and nonirradiated wounds. HLA expression on irradiated and nonirradiated wounds treated with the chitosan skin substitute was determined on days 7, 14, and 21 (Figure 7). 

**Figure 8:**
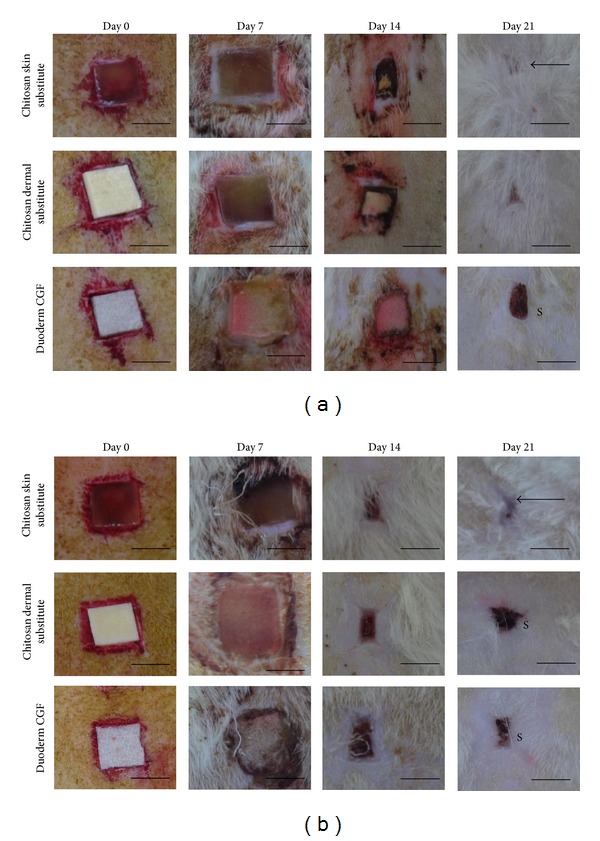
Macroscopic wound healing analysis in the irradiated (a) and nonirradiated wounds (b). On day 7, the wounds enlarged compared to day 0 due to inflammation. On day 14, the biomaterials adsorbed in (b) and the scars were clearly seen. Conversely, the biomaterials remain incorporated in (a). On day 21, a few wounds showed incomplete epithelialization presented with scab. The arrows showed the wound area. Scale bar 1 cm. (S: Scab).

From physical observation, chitosan dermal substitute and chitosan skin substitute contributed to the acceleration of healing processes both in irradiated and nonirradiated full-thickness wounds with no pus, no haemorrhages, scant exudates, no odour, and no fluid accumulation while remaining in place (adhered to the wound) and retaining their flexibility (elasticity with motion) as shown in Tables 1 and 2. The events of wound healing were macroscopically summarized in Figure 8.

## 4. Discussion

Radiation of rat skin at a high dose creates an ulcer that exhibits impaired wound repair. Histologically, irradiated skin has increased pigmentation, thickening, and fibrosis of the skin as well as alterations in sebaceous gland function. The resulting radiation damage affects the fibroblasts, keratinocytes and blood vessels and ultimately leads to skin hypoxia. Necrosis and tumorigenesis are further consequences of this impairment [11]. 

Full-thickness wounds are characterized by the destruction of the regenerative epithelial component. This type of wound heals by concurrent contraction and re-epithelialization. All excessive full-thickness wounds require skin grafting, as they show slow re-epithelialization. A lack of skin grafting can lead to extensive scarring with poor cosmetic and functional outcomes. Skin grafting creates damage at the donor site which generally heals with little scarring. The procedure causes pain at the donor site in addition to pain at the site of skin injury. Therefore, biomaterials for skin replacement are the best strategy for wound management in cases of excessive skin loss.

Our analyses have shown that the re-epithelialization in nonirradiated wounds was higher than that in irradiated wounds, but not in wounds with the chitosan skin substitute. This supports the conclusion that HFSCs, which play an important function in wound healing, do not normally respond in the healing process [12]. Re-epithelialization consists of the formation of a new epidermis by the synthesis of neokeratinocytes across a wound surface [13]. Re-epithelialization of a full-thickness wound occurs only at the edges of the wound and involves thickening and rolling beneath the edges. If the cells cannot continue to migrate across the wound bed, they build up an epithelial tongue along the edges of the wound (Figure 2) [14]. Immediately after-injury, epithelial cells start to migrate and change their phenotype. Epithelial cell mitosis is active in the migrating epithelial tongue and unwounded epidermis [15]. As a result, the migrating cells increase and the epithelial tongue develops. The migrating epidermis cells are also called the migrating epithelial tongue [16] or the migratory tongue. The cell migration terminates after the migrating epithelial tongue bridges both sides of the wound edge [17]. 

The epithelial tongue and migratory tongue distances were not significantly different between irradiated and nonirradiated wounds or between biomaterials (*P* > 0.05). This is because the measurement of the epithelial tongue formation and the migratory tongue distance were performed on day 7, which is considered a mature stage. Moreover, wound contraction had already started five days after the injury [18]. The combination of re-epithelialization and contraction increases the length of the epithelial tongue and shortens the migratory tongue distance. Therefore, an early assessment of the epithelial tongue and migratory tongue distance, for example, on day 4 is recommended to show a robust difference. 

The appearance of a scar is a clinical manifestation of the remodelling stage of healing. It involves collagen synthesis, degradation of the vascular and cellular components of scar tissue, loss of scar tissue mass, and presentation of obvious changes in the visual appearance of wound site [1]. Collagen is deposited in the scar to strengthen the wound site and is also degraded in an attempt to remodel the wound. However, there is an imbalance between collagen deposition and degradation. In certain cases, the collagen production exceeds the collagen degradation, raising a thick scar [19]. However, wound healing can be manipulated to produce more regenerative tissue and less scar formation by using skin replacement products [20]. This study demonstrated that the chitosan skin substitute and chitosan dermal substitute generate significant tissue-engineered skin with less scaring than duoderm CGF (*P* < 0.05) when used in an irradiated wound. In the nonirradiated wounds, the collagen production was interfered due to aggressive behaviour of the rats. They preferred to remove the dressing materials which cause starched to the wound bed and resulting poor collagen production. The wounds with the damaged biomaterials were not replaced with a new one, because a new biomaterial replacement will enhance wound healing and create bias to the experimental results. The irradiated rats were not aggressive compared with the nonirradiated rats. Therefore, the experimental results in the irradiated rats were consistent compared to those in the nonirradiated rats. 

If the wound therapy is appropriate, on day 14, the wound edge will be dry, the biomaterial will be incorporated into the wound, and the amount of exudate must progressively diminish. The color and odor of the wound exudate are often used as indicators of wound infections. Pus (a yellow or green exudate) is a result of excessive bacterial loads and the demise of neutrophils after they have phagocytosed debris. Signs of infection can be observed if the wound has greater than 10^5^ bacteria. As a natural antimicrobial product, the chitosan dermal substitute and chitosan skin substitute significantly decreased the colonized bacterial growth in a full-thickness wound. Moreover, both of these chitosan substitutes were created as flexible elastic foams because of their ability to conform in proportion to the applied force [21]. The ability of a biomaterial to conform and return to its nominal shape or thickness is important in support surface systems. These two chitosans have been shown to be excellent hemostatic agents both in irradiated and nonirradiated full-thickness wounds. These findings were observed as early as day 3 during secondary dressing changes, where wound hemorrhages were not present. The ability of these two chitosans to absorb excess exudates may prevent trauma to the surrounding tissue. 

K10 is a marker of differentiated epidermis. Its expression is low on the wound edge, but it is highly expressed on the migratory tongue adjacent to the wound [10]. K10 is not expressed on the wound bed as the neoepidermis is still proliferating and not fully differentiated or in a mature stage. HLA expression confirmed the mutual xenotransplantation incorporation with irradiated and nonirradiated wounds, as early as day 7 as described previously [22]. 

Rats are the most favourite wound healing animal models to recapitulate human physiology and forecast therapeutic outcomes. Rats have been widely used because of their availabilities, low cost, tractable nature, and ease of handling [23]. Additionally, established broad knowledge and promising results based on rat wound healing earned from previous studies are the main reason for rat utilizing. Inspired by the previous good results of the inflammatory evaluation in the rats treated with chitosan [24], we investigated the impact of using chitosan dermal substitute and chitosan skin substitute on impaired and nonimpaired wounds. 

Irradiated rats have resulted in compromised skin integrity which compromised wound healing in irradiated tissue to lead the impede healing [25]. Meanwhile, the nonirradiated rats are fully immunocompetent. To date, human HFSCs cultured into chitosan have never been used to repair the wound in immunocompetent model. There are accumulative evidence of immunocompetent host tolerance to xenogenic fibroblasts, mesenchymal, or epithelial cells and they survived in the host for a few weeks [26–28]. The long-term survival of xenogenic cells and their proliferation up to four months in the fully immunocompetent host without sign of immune rejection have also been reported [29]. Fibroblasts are mostly immunologically toleranst [30]. Xenogenic fibroblasts have less tendency to tissue rejection as they expressed HLA without inducing T-cell proliferation [31]. Bone marrow stem cells and mesenchymal stem cells (MSCs) have shown the potential *in vivo* immune modulation and immune privileged properties [32–35]. Additional studies suggest that MSCs derived from human or animal express costimulatory antigens which results in immunoprivileged [27, 36, 37]. As none of the rats died of infection or presented with complication particularly at the wound site in the nonirradiated rats, HFSCs-chitosan constructs are suggested less or not prone to tissue rejection. The HFSCs resulted in immunoprivileged which MSCs have done in cell or tissue therapy. Moreover, the macroscopic image and physical observation of wounds have shown the comparable inflammatory response both in the irradiated or nonirradiated wounds. Since the rats were immunocompetent, the positive results are predicted due to the rat skin as an immunoprivileged site.

## 5. Conclusion 

The radiation procedure has leaded the full-thickness wounds to have impaired healing. Direct observation of temporary hair loss on irradiated skin was present. In addition, histology analysis has proved anatomical changes of sebaceous gland as well as hair follicles. The use of chitosan dermal substitute and chitosan skin substitute has proven to accelerate full-thickness wound healing in irradiated rats as they significantly decreased the size of scar. The physical observation of wound assessment has shown that both of chitosan substitutes are ideal matrices for primary dressing. The use of HFSCs-chitosan construct on immunocompetent rat has shown their potential *in vivo* immune modulation and immune privileged properties. 

## Figures and Tables

**Table 1:** Physical observation of irradiated wounds after treatment with three biomaterials.

Assessment (Score)	Mean ± SEM		
	Day 7	Day 14	Day 21
Infection-pus			
Chitosan skin substitute	1.0 ± 0	1.0 ± 0	1.0 ± 0
Chitosan dermal substitute	1.0 ± 0	1.0 ± 0	1.0 ± 0
Duoderm CGF	1.0 ± 0	1.0 ± 0	1.0 ± 0
Hematoma			
Chitosan skin substitute	1.0 ± 0	1.2 ± 0.20	1.0 ± 0
Chitosan dermal substitute	1.0 ± 0	1.0 ± 0	1.0 ± 0
Duoderm CGF	1.0 ± 0	1.0 ± 0	1.0 ± 0
Exudation			
Chitosan skin substitute	3.0 ± 0	3.0 ± 0	3.0 ± 0
Chitosan dermal substitute	3.0 ± 0	3.0 ± 0	3.0 ± 0
Duoderm CGF	3.0 ± 0	3.0 ± 0	3.0 ± 0
Odor			
Chitosan skin substitute	3.0 ± 0	3.0 ± 0	3.0 ± 0
Chitosan dermal substitute	3.0 ± 0	3.0 ± 0	3.0 ± 0
Duoderm CGF	3.0 ± 0	3.0 ± 0	3.0 ± 0
Flexibility			
Chitosan skin substitute	2.4 ± 0.24	2.4 ± 0.24	3.0 ± 0
Chitosan dermal substitute	2.2 ± 0.20	2.4 ± 0.24	2.4 ± 0.24
Duoderm CGF	2.00 ± 0	2.4 ± 0.24	2.4 ± 0.24
Adherence			
Chitosan skin substitute	2.8 ± 0.20	1.8 ± 0.73	0.6 ± 0.60
Chitosan dermal substitute	3.0 ± 0	1.8 ± 0.73	1.2 ± 0.73
Duoderm CGF	2.4 ± 0.24	2.4 ± 0.60	1.8 ± 0.73
Fluid accumulation			
Chitosan skin substitute	3.0 ± 0	3.0 ± 0	3.0 ± 0
Chitosan dermal substitute	3.0 ± 0	3.0 ± 0	3.0 ± 0

The means were not significantly different between biomaterials (*P *> 0.05).

**Table 2:** Physical observation of nonirradiated wounds after treatment with three biomaterials.

Assessment (Score)	Mean ± SEM		
	Day 7	Day 14	Day 21
Infection-pus			
Chitosan skin substitute	1.0 ± 0	1.0 ± 0	1.0 ± 0
Chitosan dermal substitute	1.0 ± 0	1.0 ± 0	1.0 ± 0
Duoderm CGF	1.0 ± 0	1.0 ± 0	1.0 ± 0
Hematoma			
Chitosan skin substitute	1.0 ± 0	1.3 ± 0.33	1.0 ± 0
Chitosan dermal substitute	1.0 ± 0	1.3 ± 0.33	1.0 ± 0
Duoderm CGF	1.0 ± 0	1.3 ± 0.33	1.0 ± 0
Exudation			
Chitosan skin substitute	3.0 ± 0	3.0 ± 0	3.0 ± 0
Chitosan dermal substitute	3.0 ± 0	3.0 ± 0	3.0 ± 0
Duoderm CGF	3.0 ± 0	3.0 ± 0	3.0 ± 0
Odor			
Chitosan skin substitute	3.0 ± 0	3.0 ± 0	3.0 ± 0
Chitosan dermal substitute	3.0 ± 0	3.0 ± 0	3.0 ± 0
Duoderm CGF	3.0 ± 0	3.0 ± 0	3.0 ± 0
Flexibility			
Chitosan skin substitute	2.00 ± 0	2.7 ± 0.33	2.6 ± 0.24
Chitosan dermal substitute	1.8 ± 0.20	3.0 ± 0	2.6 ± 0.24
Duoderm CGF	2.4 ± 0.24	3.0 ± 0	2.4 ± 0.24
Adherence			
Chitosan skin substitute	3.0 ± 0	2.0 ± 1.0	3.0 ± 0
Chitosan dermal substitute	3.0 ± 0	2.0 ± 1.0	3.0 ± 0
Duoderm CGF	2.4 ± 0.60	2.0 ± 1.0	3.0 ± 0
Fluid accumulation			
Chitosan skin substitute	2.8 ± 0.20	2.0 ± 1.0	3.0 ± 0
Chitosan dermal substitute	3.0 ± 0	2.0 ± 1.0	3.0 ± 0

The means were not significantly different between biomaterials (*P *> 0.05).
